# Direct visualization of the effect of DNA structure and ionic conditions on HU–DNA interactions

**DOI:** 10.1038/s41598-021-97763-w

**Published:** 2021-09-16

**Authors:** Szu-Ning Lin, Remus T. Dame, Gijs J. L. Wuite

**Affiliations:** 1 Leiden Institute of Chemistry, Leiden University, Leiden, The Netherlands; 2 Department of Physics and Astronomy, Vrije Universiteit Amsterdam, Amsterdam, The Netherlands; 3 Centre for Microbial Cell Biology, Leiden University, Leiden, The Netherlands; 4 LaserLaB Amsterdam, Vrije Universiteit Amsterdam, Amsterdam, The Netherlands

**Keywords:** Biochemistry, Biophysics

## Abstract

Architectural DNA–binding proteins are involved in many important DNA transactions by virtue of their ability to change DNA conformation. Histone-like protein from *E. coli* strain U93, HU, is one of the most studied bacterial architectural DNA–binding proteins. Nevertheless, there is still a limited understanding of how the interactions between HU and DNA are affected by ionic conditions and the structure of DNA. Here, using optical tweezers in combination with fluorescent confocal imaging, we investigated how ionic conditions affect the interaction between HU and DNA. We directly visualized the binding and the diffusion of fluorescently labelled HU dimers on DNA. HU binds with high affinity and exhibits low mobility on the DNA in the absence of Mg^2+^; it moves 30-times faster and stays shorter on the DNA with 8 mM Mg^2+^ in solution. Additionally, we investigated the effect of DNA tension on HU–DNA complexes. On the one hand, our studies show that binding of HU enhances DNA helix stability. On the other hand, we note that the binding affinity of HU for DNA in the presence of Mg^2+^ increases at tensions above 50 pN, which we attribute to force-induced structural changes in the DNA. The observation that HU diffuses faster along DNA in presence of Mg^2+^ compared to without Mg^2+^ suggests that the free energy barrier for rotational diffusion along DNA is reduced, which can be interpreted in terms of reduced electrostatic interaction between HU and DNA, possibly coinciding with reduced DNA bending.

## Introduction

The way in which a protein interacts with DNA determines the kinetics of this interaction and the motion of this protein on DNA. The motion of a protein on DNA can be described in physical terms as (rotation-coupled; due to the helical nature of double stranded DNA) 1D diffusion combined with 3D excursions such as hopping, jumping, or intersegmental transfer^1,2^. 3D excursions strongly enhance apparent 1D diffusion rates and are key to an effective target searching of sequence-specific DNA binding proteins^1,2^. The motion of a protein along DNA is related to charge^3,4^, DNA conformation^5,6^ and possibly protein conformation^7^. Some proteins can bind to DNA in more than one conformation. These different conformations of a protein can occur in solution before binding to DNA, for example as a consequence of varying physico-chemical conditions. Binding to DNA can then lead to multiple diversely structured protein–DNA complexes. Distinctive conformations can also arise during the matching of the protein structure to a DNA substrate^8–10^. Hence, understanding the potential conformations of a protein, what drives changes in conformation, and what is the structure of the resultant protein–DNA complex is key to understanding the kinetics of protein–DNA interactions and protein function.

Typical architectural DNA–binding proteins in bacteria are the nucleoid-associated proteins (NAPs). Members of this family of proteins are believed to be instrumental in compacting and organizing the genome, as well as to hinder or facilitate genome transactions^11–13^. Many of these proteins exhibit multiple ways of binding to DNA, which results in different protein–DNA complex structures. For instance, H-NS exists in two conformations (open or closed), which upon binding result in formation of either a lateral protein filament along DNA or bridges between DNA segments^14,15^. FIS also exhibits two modes of DNA binding: either it bends DNA or forms bridges between DNA segments^16,17^. HU similarly exhibits two distinct modes of binding to DNA: at low binding density HU proteins bend DNA and at high binding density they form protein filaments along DNA^18–22^. Which mode of binding predominates often depends on the local protein concentration which is affected by the ionic conditions^20^. Other factors such as the crowding environment^23^, pH, and temperature^24^ have also been shown to affect the type of protein–DNA complexes formed. In the case of H-NS it was demonstrated that a switch between modes of binding results from a conformational change in the protein mediated by a change in ionic strength^14,15^. However, whether changes in physico-chemical conditions affect the type of complexes formed and whether this is due to changes in protein conformation or ‘simply’ due to effects on electrostatic protein–DNA interactions is unclear for most architectural DNA–binding proteins.

In this study, we investigate HU, a conserved DNA–binding protein found in most bacteria. This protein exists as a heterodimer or homodimer in *Escherichia coli* (*E. coli*)*,* with two genes encoding the two different HU subunits (${\text{HU}}_{{\upalpha }}$ and ${\text{HU}}_{\upbeta }$) ^25^. In other bacterial species, HU often exists as a homodimer. As outlined above HU binding is known to occur in two distinct modes as a function of protein concentration^19,22,26^. The DNA binding properties of HU do not depend on specific DNA sequences, but rather on DNA bending/intrinsic curvature, evidenced as a preference for AT-rich DNA^27–29^. Binding of HU to DNA is tension-dependent: application of force induces protein dissociation^29,30^. Moreover, binding of HU to DNA is sensitive to ionic strength: increasing the concentration of NaCl or K-glutamate reduces the affinity of HU for DNA^30^.

The binding of HU to DNA and its motion along DNA have been investigated in single-molecule fluorescence imaging studies both in vitro ^31^ and in vivo^32^, yielding information on binding and diffusion kinetics. The diffusion coefficients measured in these studies are not very similar: the diffusion coefficient is five times higher in vitro than in vivo (0.5 μm s^−1^ in vitro vs 0.1 μm s^−1^ in vivo). This discrepancy is attributed to different ionic conditions and the complexity of the in vivo medium with the numerous macromolecules present in the cytoplasm resulting in crowding, which likely influences the DNA binding properties of HU^33^. However, both studies underline the dynamic nature of the bound HU on DNA.

To elucidate the dynamics of HU–DNA interactions we here investigate (1) the effect of Mg^2+^ on the DNA binding behavior of HU and (2) the effect of different structural states of DNA using applied tension as a parameter. Using confocal microscopy combined with optical tweezers in a microfluidic system^34,35^, we visualize and characterize the binding of individual HU dimers on DNA. Our studies reveal that the binding of HU is tension-dependent and that HU stabilizes double stranded DNA during overstretching. The motion of HU along DNA is dramatically different depending on whether Mg^2+^ is present or not. With 8 mM Mg^2+^, the diffusion coefficient is in the order of 0.1 μm s^−1^, but the diffusion coefficient is more than an order of magnitude smaller in the absence of Mg^2+^. The impact of magnesium ions on HU sliding along DNA is significant because Mg^2+^ is also present in variable amounts in vivo*,* which might be implied in regulatory roles of HU.

## Results

**Figure 1:**
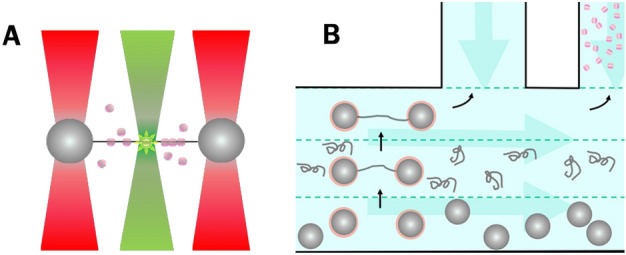
Schematic illustrating the experimental layout of an experiment using confocal optical tweezers. (**A**) Alexa555-labelled HU proteins (pink spheres) are excited at 532 nm. Fluorescence emission is measured at 555 nm. (**B**) A top view of a microfluidic sample chamber with laminar flow. DNA was captured between beads, and HU–DNA complexes were assembled inside the protein channel and in the presence of flow. The measurements were carried out inside one of the two top channels without flow.

For our studies of HU–DNA interaction, we use an integrated optical tweezers/confocal microscopy instrument to permit concurrent manipulation of DNA and to visualize DNA-bound fluorescently labelled proteins (Fig. 1A). The microfluidic sample chamber permits efficient repeated stepwise capture and assembly of protein–DNA complexes between beads (Fig. 1B), followed by mechanical characterization and fluorescent microscopy visualization^34,35^.

### HU protein binds DNA under high tension

**Figure 2:**
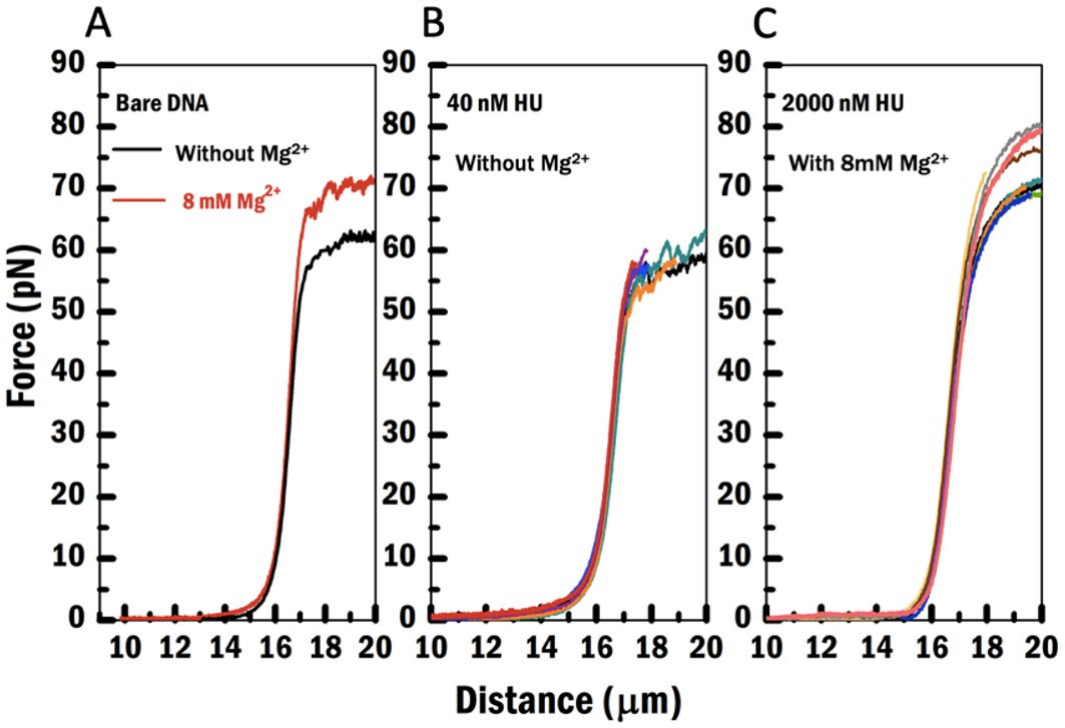
Force-Distance curves of (**A**) bare dsDNA without and with 8 mM Mg^2+^ containing buffer, (**B**) multiple representative HU–DNA complexes without Mg^2+^ at a concentration of 40 nM HU, and (**C**) multiple representative HU–DNA complexes with 8 mM Mg^2+^ at a concentration of 2000 nM HU. The data shown in (**A**) were acquired in a protein-free channel, that of (**B**) and (**C**) in a channel containing HU protein.

In order to determine the tension dependent DNA binding characteristics of HU on DNA and to characterize the impact of Mg^2+^ on this, we determined the force-extension behavior of individual dsDNA molecules as a function of HU concentration, both in presence and absence of 8 mM Mg^2+^. First, we performed a control experiment on bare $\lambda$ DNA without and with 8 mM Mg^2+^ (Fig. 2A). The force-distance (FD) curves of bare dsDNA molecules both without and with 8 mM Mg^2+^ exhibit a saw-tooth shape at high tension indicative of dsDNA unpeeling during overstretching. The overstretching occurs at 58.6 ± 0.5 pN without Mg^2+^ (N = 98, containing 50 mM NaCl, 10 mM Tris pH 7.5, 1 mM DTT) and 67.8 ± 0.4 pN with 8 mM Mg^2+^ (N = 92, containing 50 mM NaCl, 10 mM Tris pH 7.5) (Table 1). A similar effect of Mg^2+^ on force-extension behavior of bare DNA has been reported before^36^. It is thought that this effect can be attributed to neutralization of the backbone charge of DNA by Mg^2+^ resulting in reduced intramolecular repulsion^36^. Hence it effectively stabilizes the helical twist of the dsDNA and consequently a higher force is required to reach overstretching.

**Table 1:** The occurrence of saw-tooth vs smooth features on force-extension curves at high tension.

HU concentration (nM)	Saw-tooth overstretching tension (pN)	Smooth overstretching tension (pN)	Saw-tooth vs smooth N
**Without Mg**^**2+**^			
0	58.6 ± 0.5	–	98:0
40	58 ± 1	62 ± 1	25:19
400	64 ± 5	81 ± 1	6:19
2000	–	75 ± 1	0:25
**With 8 mM Mg**^**2+**^			
0	67.8 ± 0.4	–	92:0
40	64 ± 1	68 ± 1	36:19
400	–	79.7 ± 0.4	0:25
2000	–	73 ± 1	0:16

Error are SEM (standard error of the mean).

In contrast, HU–DNA complexes exhibit distinct features depending on the HU concentration (Fig. 2B,C). Smooth overstretching becomes gradually more prevalent when the HU concentration is increased. Moreover, the ratio of smooth overstretching and saw-tooth overstretching changed differently depending on whether Mg^2+^ was present or not (Table 1). The transition from sawtooth to smooth overstretching occurs at lower HU concentration when Mg^2+^ is present. Note the complete absence of saw-tooth shaped curves at HU concentrations 400 nM and higher in the presence of Mg^2+^. This observation suggests that Mg^2+^ increases the binding affinity of HU at high tension and the bound HU suppresses the unpeeling of DNA. In addition, we found that the overstretching/unpeeling occurs at even higher tension at HU concentrations above 400 nM compared to the bare DNA with 8 mM Mg^2+^. These results suggest that HU stabilizes double stranded DNA, in line with earlier DNA unzipping experiments^37^, and remains bound to DNA at high tension. At high HU concentration (2000 nM), the bound proteins change the overstretching transition regardless of the salt condition, i.e. smooth overstretching appears both without and with 8 mM of Mg^2+^ (Table 1). Note that there is some variability in the height of the overstretching force, which might be due to the formation of HU filaments to different extents at these very high HU concentrations which, in turn, hampers overstretching of DNA.

**Figure 3:**
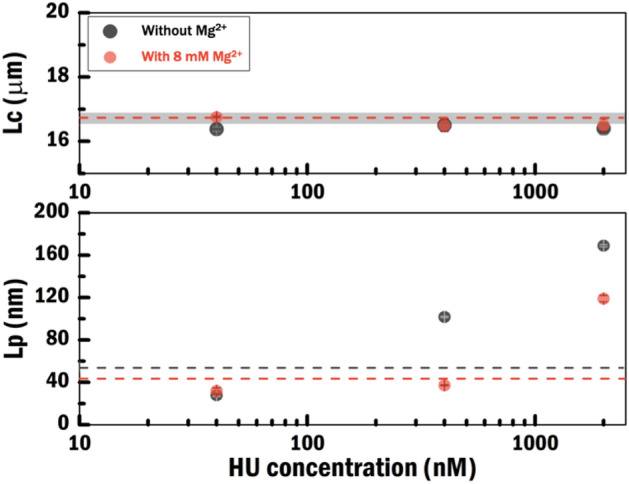
Contour length (Lc) and persistence length (Lp) as a function of protein concentration. Grey and red solid circles represent values obtained for the HU–DNA complexes measured without and with 8 mM Mg^2+^, respectively. Thin dashed lines represent the values without HU protein. Lc values without HU protein are overlapping in both salt conditions. Thick shaded line represents the error bar. Data points for a concentration of 40 nM HU were obtained by fitting force-extension curves from both Alexa-555-labelled and wild-type HU–DNA complexes. All data points in the figure were obtained by fitting a Gaussian function to Lp and Lc values obtained from extensible-WLC fitting to FD traces (0, 40, 400, 2000 nM HU conditions with N = 85, 9, 25, and16 curves, respectively).

Next, to quantify the effect of Mg^2+^ on HU–DNA complexes, we determined the persistence length (Lp) and contour length (Lc) from force-extension curves as a function of HU concentration in both salt conditions, (Fig. 3; Supplementary SI Table 1). Lc is independent of the HU concentration both without and with 8 mM Mg^2+^ (Fig. 3, top). In contrast, it was found that the Lp, is dependent on the HU concentration in both experimental conditions. Lp is reduced compared to bare DNA at low HU concentration due to HU binding as expected^22,26^ (Fig. 3, bottom). Lp increases compared to bare DNA at high HU concentration. The dashed line in this figure indicates the Lp of bare DNA. The decrease in Lp is due to HU-induced bending of DNA resulting in compaction, whereas the increase in Lp corresponds to HU–DNA filament formation. However, a lower magnitude of the increase in Lp was observed in the condition with 8 mM Mg^2+^. This is in agreement with earlier observations that increasing ionic strength reduces HU binding affinity^30^. Overall our results agree with earlier observations^22,26,33^ and indicate switching between two modes of binding depending on HU concentration.

### Mg^2+^ changes the affinity of HU binding to DNA

**Figure 4:**
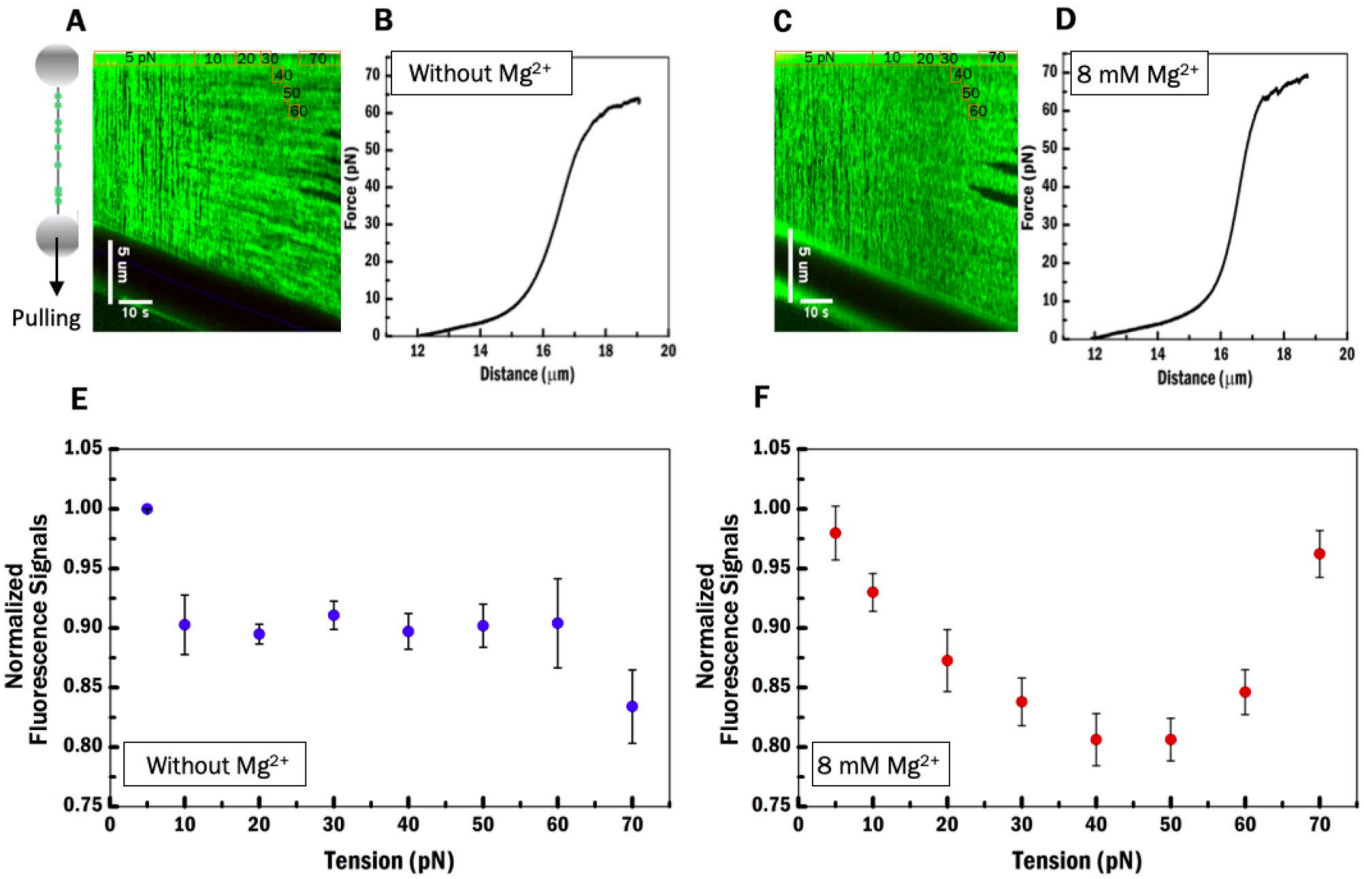
Visualization of fluorescently labelled HU protein along DNA at different tensions with and without 8 mM Mg^2+^. (**A**) Left is an illustration of a DNA pulling experiment. (**A**, **B**) Show a kymograph of fluorescence data and a FD curve obtained for the same molecule as the adjacent kymograph data without Mg^2+^. (**C**, **D**) Show a kymograph of fluorescence data and a FD curve obtained for the same molecule as the adjacent kymograph data with 8 mM Mg^2+^. Experiments were recorded in 40 nM Alexa555-HU protein channel. (**E**, **F**) Show the normalized fluorescence signals of the HU–DNA complex within 10 pN tension ranges without or with 8 mM Mg^2+^, respectively. Numerical values are an average of five experiments. The error bars represent the SE (standard error).

In order to determine the nature of the effect of magnesium cations on HU–DNA interaction, we performed experiments using fluorescently labelled HU to visually track bound protein on DNA. For this purpose, we fluorescently labelled a ${\text{HU}}_{\upbeta }$ mutant with a single cysteine at position 43 with Alexa555 (“Materials and methods”). Visualization of fluorescent HU proteins on DNA yields a 1D-fluorescence intensity pattern along the contour of the DNA (Fig. 4A, left). To track changes in this pattern of time, we can obtain such traces to obtain a 2D-fluorescence intensity pattern, called a kymograph (Fig. 4A (right),C). The kymographs with and without Mg^2+^ reveal distinct differences in binding of HU. Clusters of fluorescent signals of proteins appear along the timeline in the condition without Mg^2+^ and at tension above 10 pN, while no evidence for clusters is found with 8 mM Mg^2+^. In our earlier studies^33^ we showed that HU dimers bind DNA with high cooperativity (both in the absence and presence of 8 mM Mg^2+^) when there is no tension on the DNA. Similar binding behaviour of HU is expected at tension under 10 pN since the extension of the DNA is shorter than Lc below that force and thus somewhat comparable to the condition in the TPM experiment. Protein cluster formation is pronounced at higher tension without Mg^2+^, while protein cluster formation is suppressed by Mg^2+^. At high tension, the straight DNA molecule acts against the bending force of HU proteins^22,29^ and thus the bending mode of the bound HU proteins is suppressed. Multiple HU dimers together can form filaments along DNA^22,26,38^ and such filaments appear as fluorescent clusters along the DNA. As the stretching force increases, the distance between the sites at which HU proteins are bound to DNA, also increases. This may reduce the interaction between adjacent HU proteins, mediated by the opposite surface charge of HU ‘arms’ and ‘body’ ^39^ (Supplementary SI Fig. 1a). These dimer–dimer interactions, which occur in addition to the HU–DNA interaction, would cause cooperativity in filament formation. Without Mg^2+^ a part of the ‘body’ of the dimer could interact stronger with parts of the ‘arms’ of the other HU dimers promoting filamentation. Mg^2+^ could neutralize the negative surface of the HU dimer at the ‘body’ (Supplementary SI Fig. 1a), hence reducing the attraction between individual HU dimers.

Some dark regions are visible at the center of the HU–DNA complex at tensions above 60 pN. These are AT-rich areas which melt forming bubbles during overstretching^40^ (Fig. 4B,D). Whereas HU can bind to single-stranded DNA (ssDNA), the binding affinity is threefold lower than that for binding double-stranded DNA^39,41,42^. Most importantly anti-cooperativity (ω ~ 0.4) is observed^43^, explaining the lack of protein binding. The high fluorescent signal outside the dark region of the DNA suggests that during overstretching, DNA forms bubbles but does not peel from the ends, in a HU–DNA complex.

The normalized fluorescence signals collected for eight tension ranges (0–5, 5–10, 10–20, 20–30, 30–40, 40–50, 50–60, 60–70 pN) indicate the amount of bound proteins on DNA in each range. The amount of bound HU on DNA is affected by DNA tension and by the presence of 8 mM Mg^2+^ (Fig. 4F). Without Mg^2+^, HU binds to the DNA in a tension independent manner at tensions between 10 and 60 pN (Fig. 4E). Our observations in the absence of Mg^2+^ and under 10 pN agree with the results of Xiao et al. that HU proteins dissociate from the DNA as a consequence of the tension applied to the DNA^29^. The normalized fluorescence signals decreased upon increasing tension up to 50 pN in the presence of Mg^2+^. HU dimers tend to dissociate from the DNA and/or are less likely to bind to DNA. However, under these conditions (i.e. with Mg^2+^) the normalized fluorescence signals increase again at tensions above 50 pN indicating that the DNA binding affinity of HU increases. This suggests that structural changes induced in the DNA favor HU dimer binding and stabilize bound protein. HU is possibly using another configuration to bind on DNA due to Mg^2+^. The high fluorescence intensity could also be attributed to HU binding at forks, nicks and the junctions between single and double-strand DNA, which have been reported as high affinity targets of HU in earlier studies^44^, HU dimer with a different configuration in Mg^2+^ might have even higher affinity to these structures. Note that the DNA states at 70 pN in presence and absence of Mg^2+^ are different; dsDNA starts to melt at lower tension in absence of Mg^2+^ and hence the normalized fluorescence signals are lower.

### Mg^2+^ increases HU mobility on DNA

**Figure 5:**
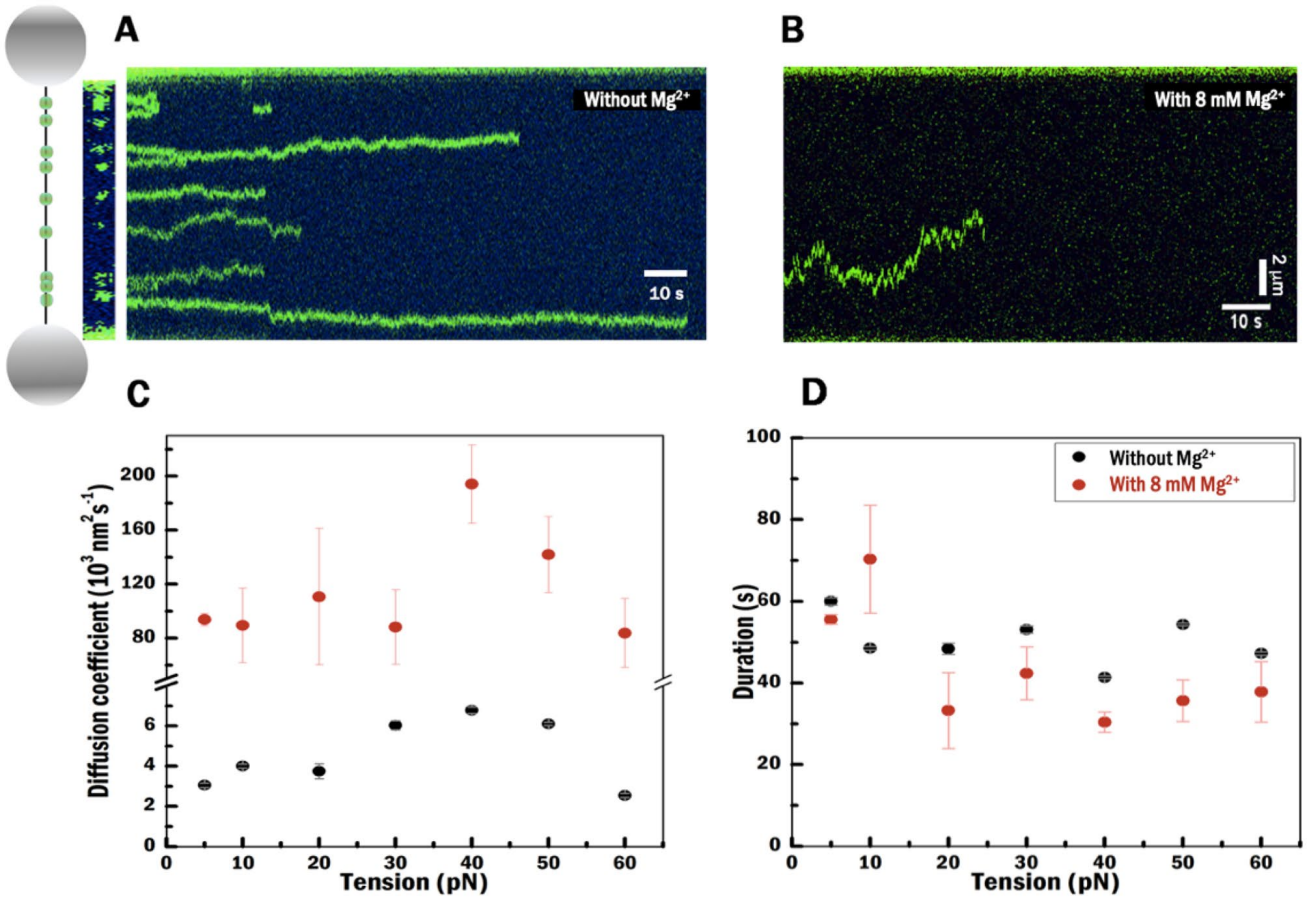
Visualization of individual fluorescently labelled HU proteins to investigate diffusion behaviour and DNA binding stability. (**A**) Illustration of the experiment and the condition where the labelled proteins are located on DNA without Mg^2+^ in the kymographs. (**B**) Example of diffusion of a single fluorescently labelled HU along dsDNA with 8 mM Mg^2+^. (**A**, **B**) Kymograph data under 20 pN. (**C**) Diffusion coefficients at a full range of tensions without (gray) and with (red) 8 mM Mg^2+^. (**D**) Shows durations at full range of tensions. (**C**, **D**) Obtained from Gaussian fitting on 1000-bootstrap resamples (raw data N > 14). Error bar in the standard error of the mean.

To further investigate the DNA binding dynamics of HU protein as a function of Mg^2+^ and tension, we visualized individual HU dimers bound to DNA (Fig. 5A). We incubated DNA in the protein channel with 40 nM fluorescently labelled HU, and next observed the behavior of DNA-bound HU in a protein-free channel. The most evident difference when comparing conditions without and with 8 mM Mg^2+^ is the amount of HU bound to DNA. Without 8 mM Mg^2+^, a five-times higher amount of proteins is bound: 854 vs 170 individual HU dimers, on multiple DNA molecules at different tensions, are bound in the first frame of Kymographs. This observation suggests that Mg^2+^ reduces the affinity of HU.

The second feature that stands out relates to the motion of the HU protein along the DNA: HU is almost immobile without Mg^2+^, while HU moves along the DNA in buffer with 8 mM Mg^2+^ (Fig. 5A,B). To quantify these differences, we determined the diffusion coefficient (D) by fitting the data to 1-dimensional mean square displacement (MSD) curves for individual proteins (N > 14 for each tension)^45^. The D values at each experimental condition are obtained from a Gaussian fitting to a 1000-bootstrap resampling (“Materials and methods”). The diffusion coefficients are roughly 30-times higher in the buffer with 8 mM Mg^2+^ on average (120 ± 20 nm^2^ s^−1^) compared to that without Mg^2+^ (5 ± 1 nm^2^ s^−1^) (Fig. 5C). This analysis indicates that Mg^2+^ enhances the mobility of HU on dsDNA. In a recent study, Leven and Levy^7^ calculated the energy barrier of HU to move on DNA, illustrating that the electrostatic interaction between protein and DNA causes an energy barrier for protein motion on the DNA. Therefore, the higher mobility of HU with 8 mM Mg^2+^ could be due to shielding of DNA from the positively charged interaction surface involving the arms and extending down the side of HU^39^ in the presence of Mg^2+^. Kamagata et al. have shown that changing DNA bending angles and the contact area between HU and DNA^31^ contribute to changes of the free-energy barrier. Without Mg^2+^ (Fig. 5C), a distinct difference in diffusion coefficient has been seen for HU bound to DNA at tensions above 30 pN (6 nm^2^ s^−1^) and at tensions below 30 pN (3 nm^2^ s^−1^). This difference could be explained by a weaker contact of the HU dimer with DNA since the ability of HU to bend is reduced by stretching DNA^31,44^. At 60 pN, DNA starts to melt (Table 1, 58 ± 1 pN) in Mg^2+^-free condition. A decreased diffusion rate (about 2 nm^2^ s^−1^) suggests that HU proteins might encounter roadblocks consisting of melted DNA bubbles hindering diffusion as has been reported for other proteins^46^.

Next, we obtained the residence time for each experimental condition applying the same method as used for determining the diffusion coefficient (“Materials and methods”). The residence time of HU exhibits no obvious dependence on tension both without and with 8 mM of Mg^2+^ (Fig. 5D). However, HU resides shorter on the DNA in the presence of 8 mM Mg^2+^ than without Mg^2+^. Our results are indicative of a higher off-rate, at forces above 10 pN, in the presence of 8 mM Mg^2+^, which is in line with the higher diffusion rate suggesting a looser interaction with the DNA.

## Discussion and conclusion

Here we investigated the effect of magnesium ions and DNA tension on the DNA binding behavior of HU. In earlier studies the effect of salt conditions on HU binding has been addressed^7,29,38,45^. However, these studies did not provide direct information on the dynamics of the interaction between HU and DNA. Recently, HU diffusion behavior has been visually recorded and measured in vitro and in vivo^32,44^. However, the specific effects of divalent cations and distinct DNA structures, invoked by the application of force, were not addressed in these studies.

In our research, we systematically investigated the interaction between HU and DNA in the presence and absence of Mg^2+^, and as a function of tension on the DNA. With 8 mM Mg^2+^, HU suppresses the tension-induced melting presumably by increasing the stability of the DNA duplex. Hence, overstretching is smooth suggesting that either s-DNA or bubbles are formed. Our fluorescence data show that Mg^2+^ increases the dissociation rate of HU protein to DNA. Moreover, Mg^2+^ enhances the diffusion of HU on DNA. Increased access to the DNA by HU could explain the structural stabilization of DNA during overstretching. Moreover, we speculate that the high tension on DNA provides HU dimers with more opportunities to bind DNA in a configuration involving low bending and quick rebinding of proteins will replace proteins that dissociate from DNA thus increasing the total amount of HU on the DNA at high tension.

We compared the diffusion behavior of HU on DNA reported by Kamagata et al.^31^ with that of our studies. Since the DNA was extended in DNA arrays used by Kamagata et al. it is reasonable to use the diffusion coefficient at 40 pN, where DNA is fully extended (~ Lc), for comparison. Interestingly, a very large difference in the diffusion coefficient is found in our study (0.007 μm^2^ s^−1^, without Mg^2+^) compared to that found earlier (0.39 μm^2^ s^−1^)^31^. Note, however, that both the salt conditions and the site of fluorescent labelling on HU differed between the two studies. Instead of NaCl in our study, K-glutamate (K-Glu) was used by Kamagata and colleagues. However, as the affinity of HU for DNA in NaCl and in K-glutamate (K-Glu) are similar^30^, that does not explain the difference in diffusion. We labelled HupB-cys43 at the $\beta$-strand, close to the center of the ‘body’, while Kamagata et al. labelled HupB-cys91, at the end of the C-terminal $\alpha$ helix at the ‘body’ (Supplementary SI Fig. 1B,C). To evaluate the possible influence of the labelling site on the binding and diffusion behavior of HU on DNA, we generated a simple model of a HU–DNA complex, based on the IHF-DNA complex (Supplementary SI Fig. 1b). In contrast to the bending induced by IHF—which is rigid—that induced by HU is flexible^22^. The IHF–DNA complex thus represents a lowly populated, extreme conformation. In this extreme conformation, position 43 is at a distance of 4 $\AA$, and position 91 at a distance of 10 $\AA$ from DNA. However, in the case of less bent DNA this distance increases, with HupB-43 being further away from the DNA in an unbent conformation. Moreover, at the C-terminus, the label at position 91 has more positional freedom increasing the possibility to interact with DNA compared to a label at position 43. It is feasible that the label at HupB-91 thus influences and reduces the interaction between protein and DNA because it is closer to DNA compared to position HupB-43. As a consequence, HU labelled at position 91 could move more freely with a larger diffusion rate. Using position 43 for labelling might therefore provide more reliable measurement of HU diffusion along DNA.

In our experimental condition with 8 mM Mg^2+^, the maximal diffusion coefficient, ~ 0.2 μm s^−1^, at 40 pN is still lower than that reported by Kamagata et al. in 50 mM KGlu^31^. At the lowest tension, 5 pN, a diffusion coefficient of 0.094 μm s^−1^ is found which is close to an in vivo result (0.14 μm s^−1^) reported earlier^32^. While the agreement is close, note that the in vivo environment is complex, with ionic conditions, interactions with other proteins and macromolecular crowding confounding the in vitro behavior in a simple buffer.

In order to investigate the mechanistic basis of the effects of different types of ions we applied the diffusion rotation-coupled equation also used by Kamagata et al.:

$$D = \frac{{k_{B} T}}{{6\pi \eta R + \left( {{{2\pi } \mathord{\left/ {\vphantom {{2\pi } {10d}}} \right. \kern-\nulldelimiterspace} {10d}}} \right)^{2} \left[ {8\pi \eta R^{3} + 6\pi \eta RR_{oc}^{2} } \right]}}e^{{ - \left( {{\varepsilon \mathord{\left/ {\vphantom {\varepsilon {k_{B} T}}} \right. \kern-\nulldelimiterspace} {k_{B} T}}} \right)^{2} }} .$$

where ${k}_{B}$, T, η, d, ${R}_{oc}$, R and ε denote the Boltzmann constant, temperature, solvent viscosity, the distance between two base pairs of DNA, the distance between DNA and the protein center, the protein radius, and free-energy barrier, respectively. This allow us to calculate effects on the free energy barrier, but also to consider possible effects on the radius of gyration of the protein. Note that Kamagata et al. used R, the radius of gyration of the protein structures corresponding to the HU–DNA complexes (PDB 1P71), and ${R}_{oc}\sim R$ to calculate the free-energy barrier (2.1 T${k}_{B}$) for diffusion. We obtained the free-energy barrier in 2.3 T${k}_{B}$ using the same R and ${R}_{oc}$ values (PDB 1P71), close to the previously reported value. We also obtained a radius, R = 2.52 nm, based on the measurement at 40 pN with Mg^2+^ and the free-energy barrier (ε) obtained from Kamagata’s work. It is possible that a larger angle between the two arms of HU yields a larger gyration radius or that the distance between DNA and protein, ${R}_{oc}$, is larger.

In the condition without Mg^2+^ HU is almost immobile. With Mg^2+^ diffusion might be enhanced due to an increase in the distance between positively charged DNA interaction surface of HU and DNA, hence reducing the friction diffusing over the DNA. Another possibility is that our observations can be explained based on the HU–DNA complex model of Hammel et al. They proposed that HU dimers can bind DNA using only one of the arms of the HU dimer to interact with the dsDNA helix^38^. This would effectively reduce the affinity of HU for DNA and promote sliding on DNA in line with our observations.

From a biological point of view, Mg^2+^ is a basic constituent of the cytoplasm. It is critical for many enzymatic processes. Bacterial pathogens are known to encounter variations in Mg^2+^ upon infecting host tissue. In these cases, environmental Mg^2+^ concentrations are low compared to the Mg^2+^ concentrations in the bacterial cytoplasm^47^. It is possible that switching between high and low Mg^2+^ concentration in such context has effects on HU affinity, DNA bending and filament formation, as seen in our experiments, which are translated in effects on expression of genes of the HU regulon. In summary, our study is a starting point to explain from a mechanistic point of view how DNA structure and Mg^2+^ cations affect HU dynamics. Further investigation of the details of how structural changes in DNA (e.g. changes in a twist, extension, conformation) correlate with HU movement (e.g. sliding, pause, or hopping) are required to be able to better understand and ultimately adequately include HU behavior in whole nucleoid models^48,49^.

## Materials and methods

### DNA construct

Bacteriophage $\lambda$ DNA (Spherotech) (47% GC content) was labelled at the cos sites using Klenow DNA polymerase exo-minus (Thermo Scientific) with 80 mM biotin-14-dATP and biotin-14-dCTP added to a mixture of 100 mM dTTP and dGTP non-modified deoxynucleotides (Thermo Scientific). The DNA product was purified using the GenElute PCR Clean-up kit (Sigma-Aldrich) and stored in TE buffer (10 mM Tris–HCl pH 8.0 and 1 mM EDTA) at 4 $^\circ \text{C}$. Further details can be found in^50^.

### Protein samples

Purification of wild-type HU protein was carried out as described before^19,22,51^. HU_A43C_ was expressed from a pRLM118 derivative^52^ (a kind gift from George Chaconas) following heat shock induced expression. The purification procedure was identical to that used for wild-type HU.

For fluorescent labeling, 0.2 mM HU_A43C_ was incubated in buffer (25 mM Tris pH 7.5, 1 mM EDTA pH 7.5, 10% glycerol, 50 mM NaCl) containing 0.8 mM DTT for 2 h at room temperature (RT) to break down disulfide bonds between cysteines. DTT was removed using a spin column (Illustra AutoSeq G-50 columns, Thermo Scientific) before introducing the dye. Fluorescent labeling was carried out in oxygen-free environment. To achieve this N_2_ gas was bubbled through the solution to deoxygenate the HU sample mixes with the dye, with tenfold excess of dye compared to protein for 4 h at RT. The labelled HU protein was purified using an SP column (1 ml). The protein was eluted at 200 mM NaCl. Alexa555-HU concentration was determined using BCA (Bicinchoninic Acid) protein assay (Thermo Scientific). The Alexa555 concentration was determined by NanoDrop. Using this information, the labeling ratio (maximally two fluorophores per HU dimer) was determined at 90%. The DNA bending ability of Alexa555-labelled HU was tested using optical tweezers and was found to be comparable to that of wild-type HU protein (Supplementary SI Fig. 2).

### Optical tweezers experiment

The experiments were performed on a commercial dual-trap optical tweezers instrument with confocal fluorescence imaging (C-trap; Lumicks). A $\lambda$-dsDNA molecule was captured in laminar flow between a pair of streptavidin-coated polystyrene microspheres (4.65 µm in diameter, Kisker) held by optical traps. The single dsDNA attached in between the beads was then moved into a protein channel for protein incubation. Force-extension experiments on protein–DNA complexes were performed following 1 min incubation. Individual HU–DNA complexes were discarded after one force-extension stretching measurement. Tension was applied on the protein–DNA complex by increasing the end-to-end distance of the dsDNA molecule at a rate of 0.2 μm s^−1^. All optical tweezer experiments were performed at RT.

Three HU protein concentrations were used: 40, 400 and 2000 nM. The protein was diluted in two types of buffers according to desired experimental conditions: buffer I (50 mM NaCl, 100 mM Tris–HCl pH 7.5, 1 mM EDTA) and buffer II (50 mM NaCl, 100 mM Tris–HCl pH 7.5, 8 mM MgCl_2_).

### Fluorescence experiment

The experiments in which the mechanical properties of HU–DNA complexes were characterized and those in which HU binding patterns along DNA were mapped, were carried out in the protein channel. The experiments in which the mobility of HU on DNA was investigated, were carried in a protein-free channel. Within the protein channel, measurements were carried out following a 1 min incubation. For measurements in the protein-free channel, protein-bound DNA molecules were moved from the protein channel to the protein-free channel following a 1 min incubation at different tensions. Translocation of the protein–DNA complex occurred at a constant rate of 0.2 μm s^−1^, with the end-to-end distance of DNA kept constant. A 532 nm excitation laser was used to excite Alexa555 labelled protein. An electronic multiplying charge-coupled device (EMCCD) camera was used for collecting signals.

For all fluorescence experiments 40 nM Alexa555-labelled HU protein was used. Each protein sample had been incubated in buffer I or II for 2 h on ice prior to the measurements.

### Data analysis

Lc and Lp were obtained from the force-extension curve of HU–DNA complexes by fitting to an extensible worm-like chain (eWLC) model using a custom-written program in Matlab^36^. Only data at forces below 30 pN were fitted.

The overstretched tension is defined as the tension corresponding to the first peak which appears at the beginning of the melting region on FD curve. In the overstretched region, the end-to-end distance of DNA increases by a large magnitude corresponding to a small amount of increase in tension. With 8 mM MgCl_2_, the bursts are suppressed in the melting region. Hence the overstretched tension is defined as the tension corresponding to a slope on the FD curve that is < 0.02.

### Quantification of fluorescence intensity

Fluorescence data was analyzed using ImageJ (NIH)^53^. The fluorescence intensity at each range of tension was normalized by dividing the total image pixels as a unit of fluorescence signal per pixel. The fluorescence signals per pixel of one trace have further been normalized to 1 by dividing the values of the signal for the highest value of the signal per pixel, for each trace.

### Determination of HU diffusion coefficient

The displacement of each protein dimer on the DNA was measured over time by recording fluorescence movies at a high frame rate (167 Hz). The corresponding trajectories (> 6 s) were analyzed with a custom-written MATLAB-based program^45^ that tracked the position of each Alexa-555-HU dimer as a function of time. The diffusion constant (D) was determined for each individual trajectory with the same analysis program using 1-dimensional mean square displacement (MSD)^35^. Further, we obtained the diffusion coefficient of each experimental condition, from a 1000-bootstraps (using Matlab commercial script) histogram fitted by a Gaussian function (using OriginLab) (Supplementary SI Figs. 3, 4). For each experimental condition at least 20 traces were used. Only traces spanning longer than 20 s and which did not cross other traces were considered. Errors are given as the standard deviation of the mean, which was divided by the square of the amount of raw traces of each condition.

### Determination of HU residence time

First, the residence times of each individual protein trajectory is collected from Kymographs. Residence times of all the trajectories were then categorized according to their measurement conditions; with or without 8 mM Mg^2+^ and at which tension. Due to the different number of trajectories were collected for different experimental conditions, 1000 bootstrap-resampling is applied (using a Matlab commercial script). The histograms resulting from the bootstrap are fitted by a Gaussian function (using OriginLab), (Supplementary SI Figs. 5, 6). For each experimental condition at least 20 traces were used. Only traces which did not cross other traces were considered. Errors are given as the standard deviation of the mean, which was divided by the square of the amount of raw traces of each condition.

## Supplementary Information

Supplementary Information.

## Data Availability

The datasets generated during and/or analyzed during the current study are available from the corresponding author on reasonable request.
