# Supplementary material for: Direct visualization of the effect of DNA structure and ionic conditions on HU–DNA interactions
Source: Sci Rep. 2021 Sep 16;11:18492. doi: 10.1038/s41598-021-97763-w (PMC8446073; doi:10.1038/s41598-021-97763-w)
Supplement: Supplementary file 1 — Supplementary Information. [file 41598_2021_97763_MOESM1_ESM.docx]

**Supplementary Information**

**Direct visualization of the effect of DNA structure and ionic conditions on HU-DNA interactions**

S. Ning Lin^1,2^, Remus T. Dame^1, 3*^, and Gijs J.L. Wuite^2,4*^

^1^ Leiden Institute of Chemistry, Leiden University, Leiden, The Netherlands

^2^ Department of Physics and Astronomy, Vrije Universiteit Amsterdam, Amsterdam, The Netherlands.

^3^ Centre for Microbial Cell Biology, Leiden University, Leiden, The Netherlands.

^4^LaserLaB Amsterdam, Vrije Universiteit Amsterdam, Amsterdam, The Netherlands.

* correspondence should be addressed to G.J.L.W (g.j.l.wuite@vu.nl) or R.T.D. (rtdame@chem.leidenuniv.nl)

**SI Table 1. The Lc and Lp values of HU-DNA in the condition without and with 8 mM Mg^2+^.**

| HU concentration | Lc | Lp | N |
| --- | --- | --- | --- |
| (nM) | (μm) | (nm) |  |
| Without Mg^2+^ | | | |
| 0 | 16.7 ± 0.2 | 54.5 ± 0.8 | 48 |
| 40 | 16.8 ± 0.2 | 32 ± 3 | 43 |
| 400 | 16.5 ± 0.2 | 101.7 ± 0.7 | 22 |
| 2000 | 16.4 ± 0.2 | 169 ± 1 | 29 |
| With 8 mM Mg^2+^ | | | |
| 0 | 16.7 ± 0.2 | 44.0± 0.3 | 74 |
| 40 | 16.4 ± 0.4 | 28 ± 1 | 47 |
| 400 | 16.5 ± 0.2 | 37.2 ± 0.5 | 25 |
| 2000 | 16.5 ± 0.2 | 119 ± 3 | 16 |


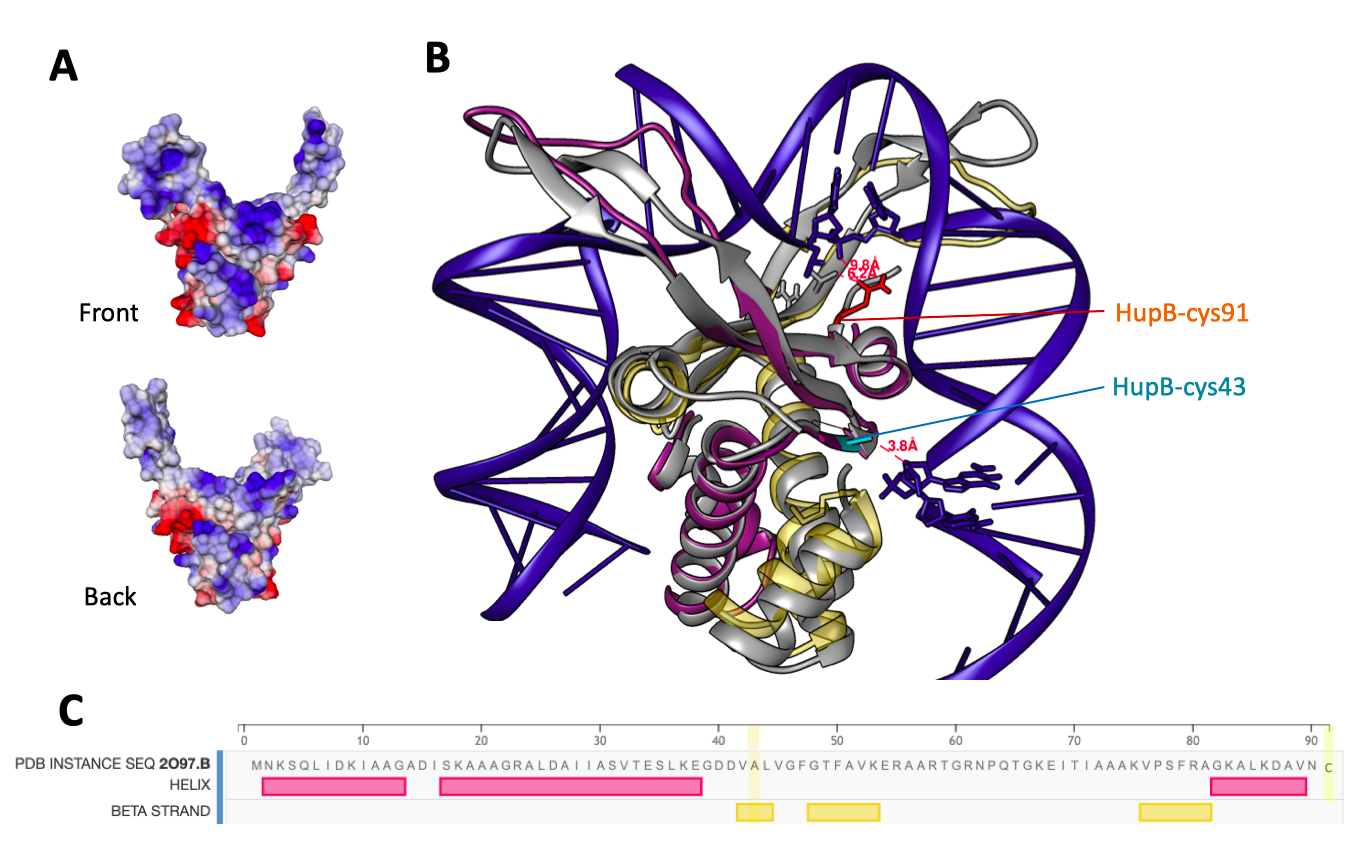


**SI Figure 1. HU surface charge and the HU-DNA bending model.** (A) The surface charges of HU protein. Homology model based on three models (PDB codes 4YEY, 4YEW and 2O97) by Modeller ^53^. Negative, neutral, and positive charges are marked in red, white, and blue indicate, respectively. (B) The structure shown is based on a homology modeling of *E.coli* IHF (PDB code 1HIF). HU$\beta$ and HU$\alpha$ are coloured in magenta and light yellow, respectively. The HU model is also aligned with an *Anabaena* HU (PDB code 1P71), in light gray, to indicate the additional amino acid at the position 91. DNA is coloured in navy blue. The distances, red dash-line, indicate the DNA elements and HU$\beta$ position 75 that are most prone to interact with the labels. The distances were analyzed by Chimera [[http://www.cgl.ucsf.edu/chimera](http://www.cgl.ucsf.edu/chimera/) ] (C) The numbering of the amino acids is according to that of E. coli HU, which is exported from PDB.


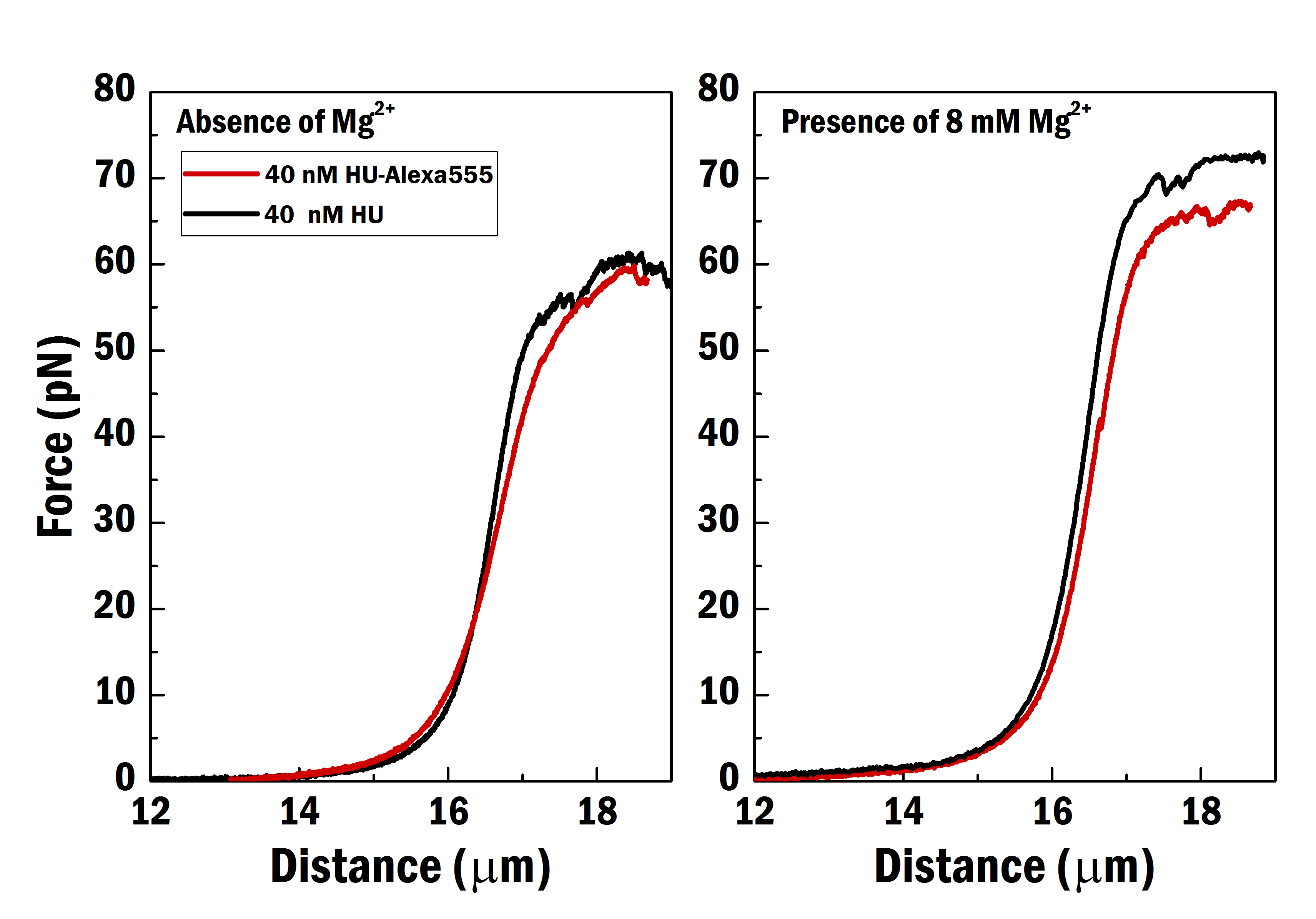


**SI Figure 2. The FD curves of Alexa555-labelled HU and wild-type HU.**


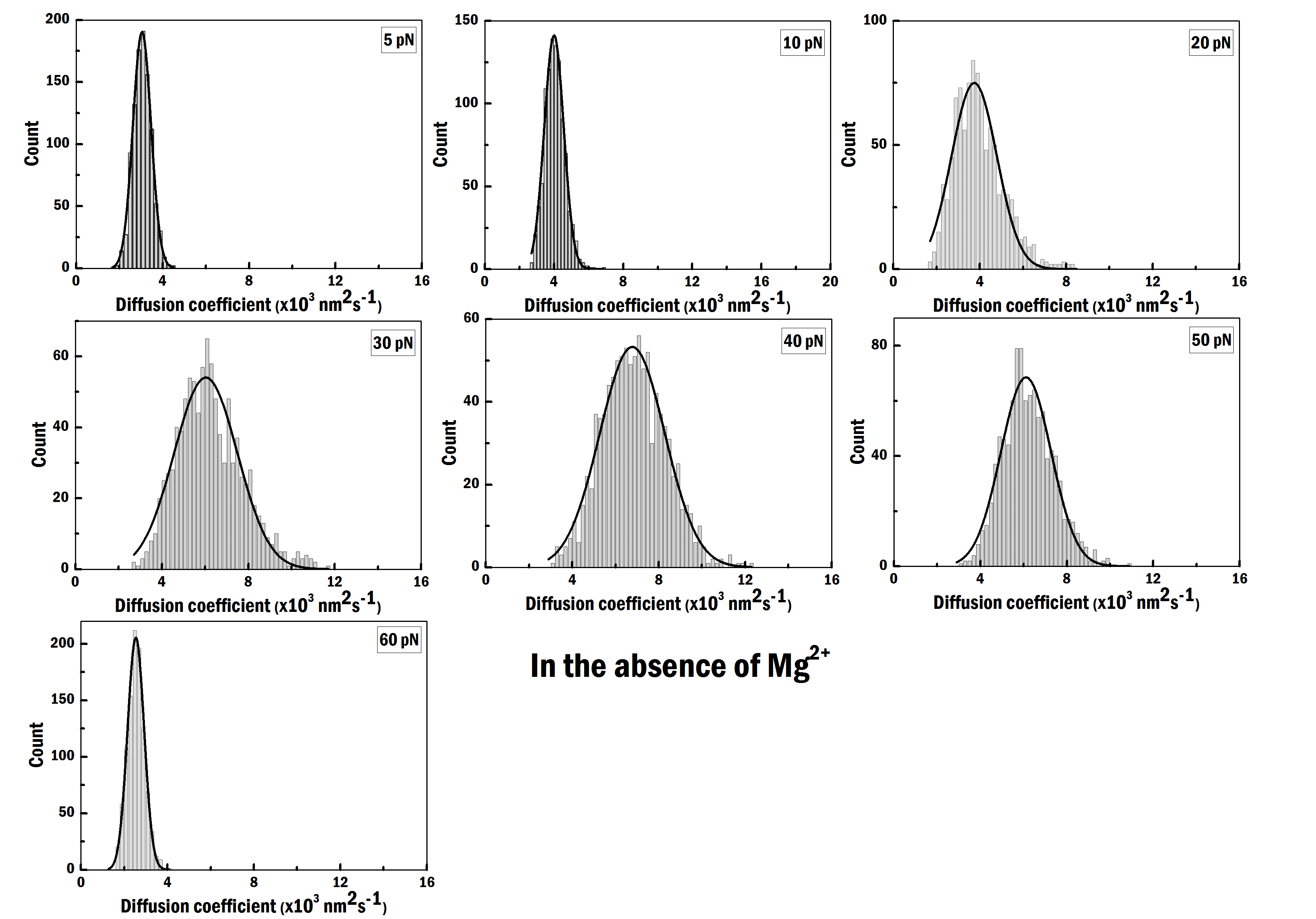


**SI Figure 3. The histogram of the diffusion coefficient in the condition without Mg^2+^.** The bin size is 200 nm^2^s^-1^.


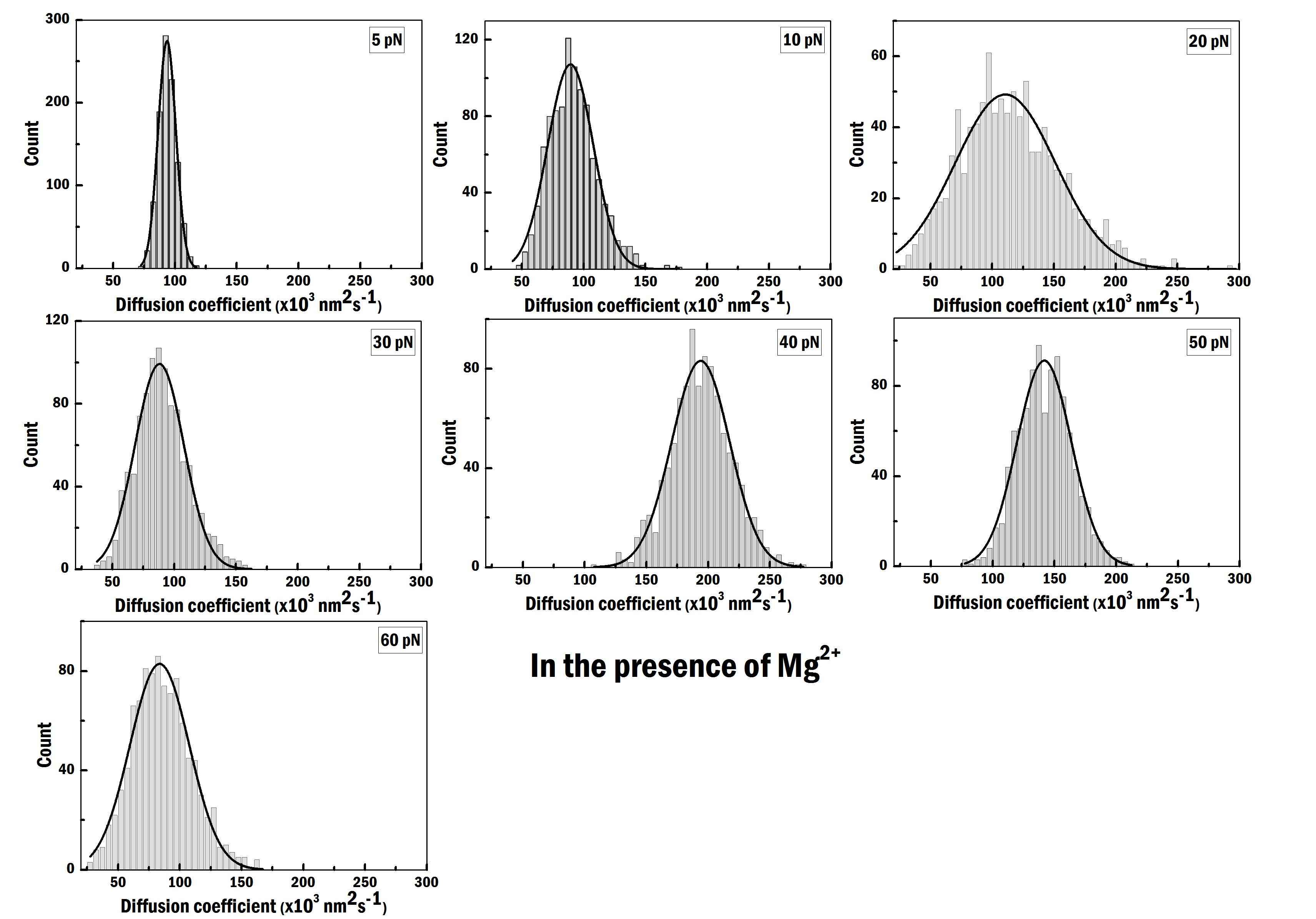


**SI Figure 4. The histogram of the diffusion coefficient in the condition with 8 mM Mg^2+^.** The bin size is 15000 nm^2^s^-1^ for data obtained at all force but 20 pN; bin size is 5000 nm^2^s^-1^ at 20 pN.


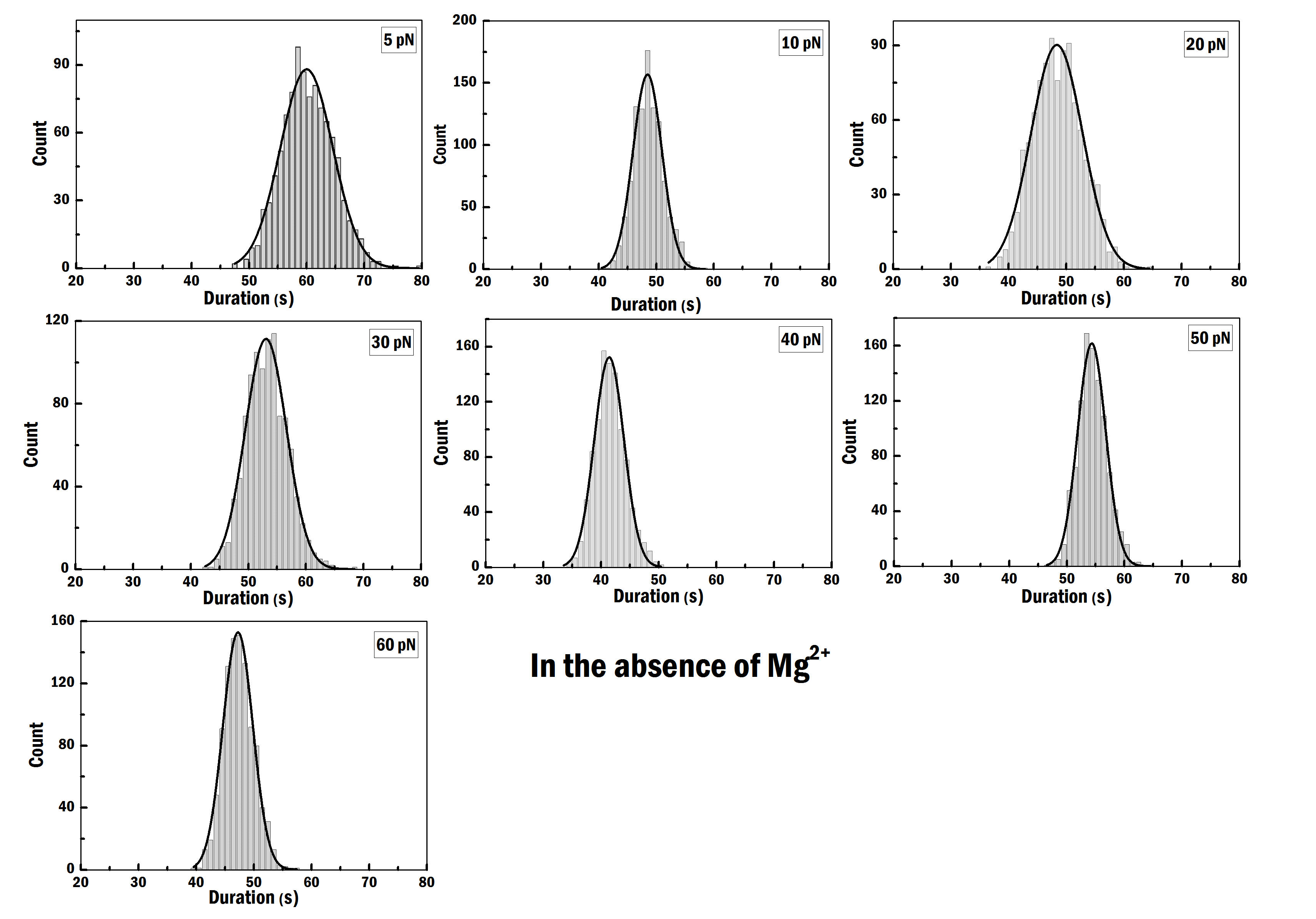


**SI Figure 5. The histogram of the duration in the condition without Mg^2+^.** The bin size is 1000 ms.


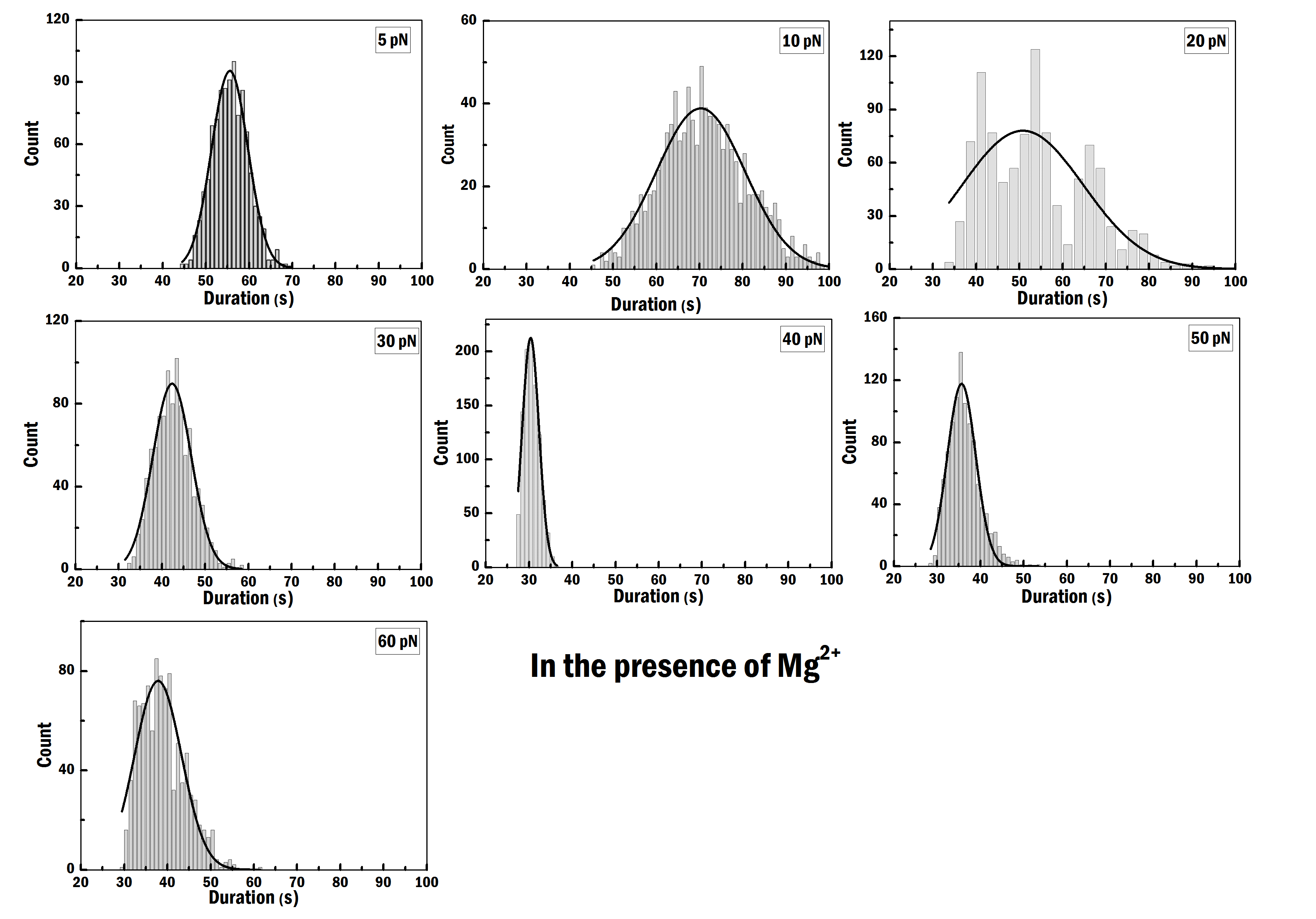


**SI Figure 6. The histogram of the duration in the condition with 8 mM Mg^2+^.** The bin size is 1000 ms.


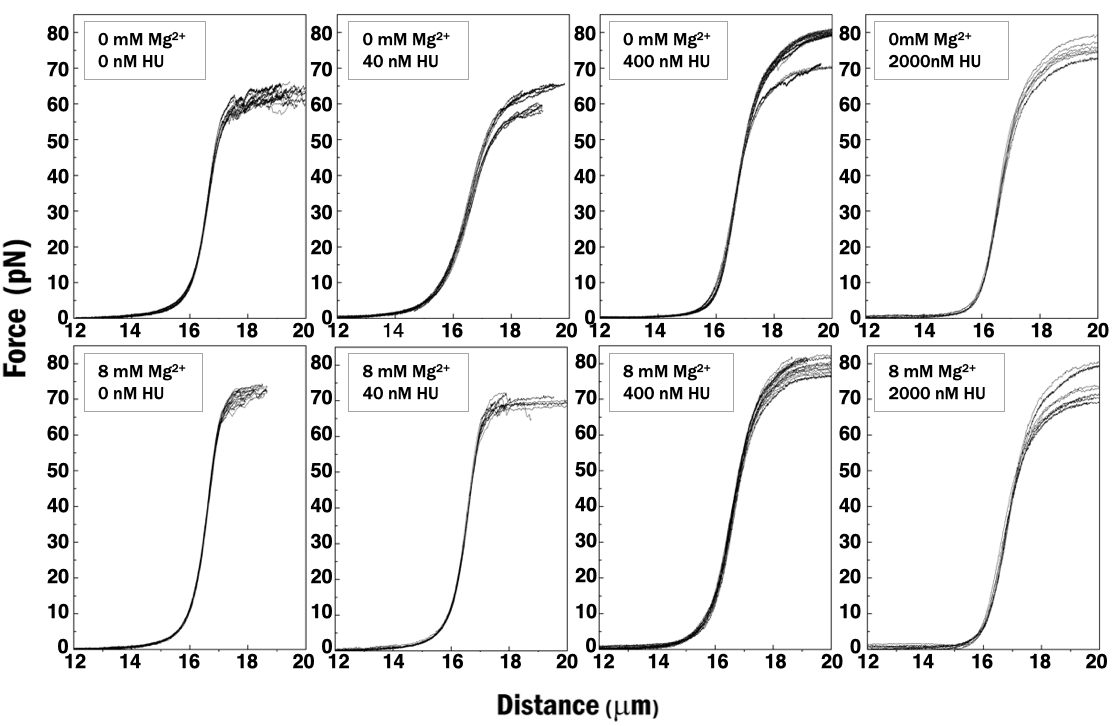


**SI Figure 7. Force-distance traces under different HU concentration within and without Mg^2+^.** The number of curves in 0, 40, 400, 2000 nM HU concentration without Mg^2+^ (N = 12, 14, 19, 8) and with Mg^2+^ (N= 17, 12, 23, 10), respectively.
